# Lectures on Typhus Fever

**Published:** 1834-07-01

**Authors:** 


*Leçons de Clinique Médicale, faites à l'Hôtel-Dieu de Paris*, par le Professeur A. F. CHOMEL; *recueillies et publiées sous ses yeux*, par J. L. GENEST, D.M.P., ancien Chef de Clinique Médicale de l'Hôtel-Dieu de Paris, &c. *(Fièvre Typhoïde.)* —Paris, 1834.

*Lectures on Typhus Fever.* By Professor CHOMEL. 8vo. pp. 548.

WE trust that, before many years have elapsed, it will be considered discreditable for a hospital physician not to have contributed his portion (or, if he cannot do that, his mite,) to the improvement of the practice of physic. The neglect of this duty might indeed be palliated, if medicine were one of the exact sciences, replete with undoubted facts, and logical deductions from them; but, as the matter stands, with doubt staring us in the face on every side, it is strange that any one should enjoy unlimited opportunities of observation and experiment for fifteen or twenty years, and yet have nothing to communicate to his less fortunate brethren. When a neighbour makes an excursion into Worcestershire, we do not wish him to write his travels; but, when the African Society send a picked man into the land of depressed crania, we expect an account of the prodigies he saw: and is not rheumatism more mysterious than Timbuctoo, and the course of fever more puzzling than the course of the Niger?

The volume before us shows that Dr. Chomel is not to be numbered among the *malingerers:* indeed, the large size of this book, giving an account of only a few years' practice in a single disease, would rather seem to demonstrate that reviewers are necessary evils, and that they are absolutely required as middle-men between author and reader.

Before we begin our abstract, we must mention an important circumstance. We concluded, from the title-page, that Professor Chomel was the sole author of this book, and that Dr. Genest was merely his shorthand writer, or amanuensis; but, when we had toiled on to page 509, and had arrived at the second paragraph of the seventh section of the seventh article, we were startled by the following note: "Ce paragraphe et l'article suivant appartiennent entièrement à M. le Professeur Chomel." We shall continue, however, to call Dr. Chomel the author, but with a slight mental reservation. We must also premise, that what the author calls *fièvre typhoïde* we call typhus fever: but that he appropriates the term *typhus* to the typhus gravior, or jail fever, exclusively.

Professor Chomel divides typhus fever into three stages, each of which lasts about a week.

*Symptoms of the First Stage.* The most usual symptoms at this period of the disease are headach, which comes on at the very commencement, and appears in almost every case; prostration of strength and stupor; diarrhœa, meteorism, and sensibility of the abdomen, especially in the right iliac region; a gurgling noise on pressure of the lower half of the abdomen; epistaxis; and, lastly, the eruption generally called typhoid eruption, which, however, scarcely belongs to this stage. If the patient should be able to sleep, his short repose is accompanied by painful dreams, which leave a lasting impression, and are confounded with his waking state, so that he thinks he has not been asleep at all: this state is called by pathologists *coma vigil.* The headach is very intense for the first week, but almost always yields at the end of this period, and sometimes sooner, to the remedies employed; but it generally lasts longer if nothing has been done. Our author does not think the colour of the tongue important as a diagnostic mark, but insists strongly on the almost constant presence of diarrhœa: some patients have one, and others fifteen or twenty liquid stools in twenty-four hours, but the most common number is from four to eight. Out of fifty-four cases in which the typhoid eruption was observed, there were but two in which it appeared during the first stage. It is very unusual for patients to die during this period. Among forty-two deaths from typhus fever in the clinical ward, but one occurred at the end of the first stage.

*Symptoms of the Second Stage.* It is generally about the seventh or ninth day from the first attack that the eruption appears which is peculiar to typhus fever, consisting of small rose-coloured spots, which disappear on pressure, from half a line to two lines in diameter, round in shape, and hardly, if at all, raised above the skin: they are scattered over the abdomen, and sometimes over the chest, and are rarely found on the thighs, arms, or forearms. These small spots are more easily distinguished in proportion to the whiteness of the skin, and are sometimes made out with difficulty in dark-coloured persons. Their number cannot be determined, because they are not all equally visible; but, in order to characterize a typhoid affection, there must be at least fifteen or twenty: if there are but two or three, nothing can be inferred from their presence. The importance of these lenticular spots has been denied, and it has been asserted that they are produced by the action of cataplasms, baths, and fomentations on the skin of the abdomen. This is obviously an incorrect explanation; for the poor rarely employ these remedies, and yet, out of seventy patients, twenty-nine had this eruption when admitted; and, moreover, it often appeared on patients in the hospital when the fomentations, &c. had not been used.

These lenticular rose-coloured spots can easily be distinguished from petechiæ and flea-bites, because in the latter kinds there is an extravasation of blood on the surface of the skin, and their colour, instead of being diminished by pressure, becomes apparently more vivid by the diminution of colour in the surrounding skin. In the typhoid spots, on the contrary, the redness disappears just as it does in erysipelas, where there is evidently congestion of blood, and reappears when the pressure ceases.

In this stage, too, occur the small vesicles called *sudamina;* ulcerations on the surface of the body, and an almost total loss of the contractile power of the muscles. This debility of the muscles sometimes extends to those which are employed in respiration, immediately endangering the life of the patient. The diarrhœa still continues, and sometimes there is hemorrhage from the intestines. The meteorism in some cases is considerably augmented, and reaches the degree of intensity to which authors have given the name of tympanitis. The abdominal pains, which are occasionally rather sharp during the first stage, disappear in the second, excepting in mild cases, where the patient preserves his senses and intellectual faculties unimpaired. Some patients die during the second stage, *i. e.* from the eighth to the fifteenth day. Of forty-two deaths in the clinical ward, nine occurred in this stage; that is to say, during the most acute stage of the disease.

*Symptoms of the Third Stage.* If the case is about to terminate in recovery, the more serious symptoms diminish in intensity. If the patient gives a quicker answer to questions, and his eyes are voluntarily turned towards the person who speaks, this first look of the patient, this expression of his physiognomy, showing that he is beginning to awake from the stupor, in which he was unconscious of all around him, is a certain sign of amendment. In other cases the coma becomes a quiet sleep, on awaking from which the patient has recovered a part of his consciousness. In the greater number of patients their movements are easier, and the difficulty of swallowing is diminished; and, though debility is still present, the patient can move himself in a way that he could not have done two or three days before. The mouth and tongue become moist; the meteorism diminishes; and the stools become yellower, more solid, and less fetid.

In unfavourable cases the stupor increases, and the alteration in the expression of the patient's face becomes more obvious; the mouth becomes drier, or, if it is moistened, it is only with the secretion of a mucous liquid, of a grey and gluey appearance, with sanious striæ and a fetid smell. Sometimes it is black or opaque, and at others has the colour of pus. The respiration becomes more and more oppressed and stertorous; and in some cases, during the last days, a crepitus can be perceived, especially at the base and posterior part of the lungs, which is occasionally succeeded by an entire absence of the respiratory murmur.

Sometimes, too, a sudden and fatal termination of the case is produced by perforation of the coats of the intestines. In fifty-five fatal cases of typhus fever, M. Louis found intestinal perforation to have taken place eight times; but, in forty-eight fatal cases at the Hôtel Dieu, it occurred only twice.

Thirty cases were bled, and there was a firm crassamentum twenty-six times, contrary to the theory of those authors who maintain that, in serious cases of fever, the blood has always lost some of its consistence.

*Post-mortem Appearances.* We shall not enter into the minutiæ with which Professor Chomel has filled more than 240 pages, but content ourselves with two or three of the more prominent points. When a patient dies during the earlier stages of the disease, the intestinal glands (Brunner's and Peyer's) are sometimes found merely swelled, and of a deeper colour than the surrounding parts, but not ulcerated; but, on the ninth or tenth day of the disease, the mucous membrane covering the *plaques gaufrés* begins to ulcerate. M. Louis, too, mentions but two cases in which he found the patches without ulceration, and there the patients had died on the eighth day of the disease. After the eighth day, he seems always to have met with ulcerated patches. When the patient dies at an advanced stage of the disease, Peyer's glands have altogether disappeared, and left nothing but ulcers to mark their place. When death takes place six weeks or two months after the first attack, the ulcers in the intestines are found to be partly healed.

These lesions are always or nearly always found, and form an essential part of typhus fever; but there are others which may be called accidental, such as redness, softening, or thickening of the mucous membrane of the stomach; enlargement of the spleen; and *ramollissement* of the liver. In almost every case the spleen is enlarged, and is frequently two, three, or even four times its natural size. The heart is often softened, and sometimes to such a degree that its muscular substance breaks down under the fingers. Our author says, that morbid appearances are rarely found in the brain, yet he gives the following table of the state of that organ in thirty-eight cases:

Injection of the membranes in 4 cases;

Œdema of the membranes in 7 cases;

General but slight ramollissement in 6 cases;

Effusion of serum in the ventricles, varying from a teaspoonful to a tablespoonful, in 12 cases;

Gritty state of the cerebral substance in 5 cases;

Anormal density in 2 cases;

Normal state in 15 cases.

*Causes.* The remote causes are unknown; but among the predisposing ones may be numbered youth, and recent residence in a great city. In 117 cases, where the age was accurately known,

| | | | |
|---|---|---|---|
| 8 | were from | 15 to 18 | years old; |
| 25 | — | 18 to 20 | — |
| 36 | — | 20 to 25 | — |
| 30 | — | 25 to 30 | — |
| 9 | — | 30 to 35 | — |
| 3 | — | 35 to 40 | — |
| 5 | — | 40 to 50 | — |
| 1 | was | 52. | |

If we add to this table the results obtained by M. Louis, and other observers, we shall find that the disease rarely occurs in persons above forty years of age, and that perhaps there is not a case on record where the patient was above fifty-five.

The majority of typhus patients admitted into the hospitals have lived but a short time at Paris, as will appear from the following table of ninety-two cases, in which this point was ascertained with precision:

| | | |
|---|---|---|
| 5 | had been in Paris | less than 1 month; |
| 10 | —— | from 1 to 3 months; |
| 9 | —— | from 3 to 6 months; |
| 21 | —— | from 6 months to 1 year; |
| 19 | —— | from 1 to 2 years; |
| 15 | —— | from 2 to 6 years; |
| 11 | —— | more than 7 years; |
| 2 | were natives of Paris. | |

Hence it appears, that sixty-four out of ninety-two, that is to say, more than two thirds, had been in Paris less than two years.

The puerperal state has likewise been considered a predisposing cause; but, so far is this from being the case, that typhus fever seems never to follow parturition. It must be recollected, that the anatomical characteristic of typhus is the lesion of the isolated or congregated follicles of the intestines, and of the mesenteric ganglia. Now, in 220 dissections performed by M. Tonnellé, at the Maternité, during the epidemic of 1829, among forty-four collected by Dr. Robert Lee, and a great number more reported in various excellent theses maintained at Paris for several years past, we have not found a single one, says Professor Chomel, in which the change in the glands of Brunner and Peyer, that we have described, is mentioned as having been found. Moreover, in fifty post-mortem examinations after typhus fever, published by M. Louis, and forty-four that took place at the Hôtel Dieu, there is not one case where the disease had followed delivery.

The section in which our author discusses the question whether typhus is contagious or not, is very sensibly written; but he concludes, as impartial physicians must do in the present state of the evidence, by declaring the point unsettled. It seems clear enough, however, that the vast quantity of miasmata generated in fever hospitals is sufficient to spread the disease; and hence it may be inferred, (if we may be allowed a mathematical mode of reasoning,) that a single case is capable of doing so likewise, but in an infinitely small degree.

Professor Chomel now gives a separate account of the various forms of typhus fever. There are five species; the inflammatory, the bilious, the mucous, the nervous, and the adynamic. We shall content ourselves, however, with giving one of his cases; it belongs to the first, or inflammatory form.

*Twenty-seventh Case.* Qurtrechy, a worker in ebony, aged twenty-two, living in Paris for the last four months, was attacked on the 21st of January, without any obvious cause, with want of appetite, weakness, and headach. He was obliged to keep his bed, and suffered from want of sleep, a slight cough, pain in his abdomen, and fever, with considerable heat during the night, and a sensation of cold during the day. The patient reported that he had vomited green and bitter matter, and had kept himself on low diet; but no other treatment had been adopted when he entered the St. Lazarus ward, No. 27, on the 25th of January, 1832.

On the sixth day of the disease, there was debility, with a difficulty of sitting upright. The patient had come to the Hôtel Dieu supported by two companions. His consciousness was perfect; his tongue moist, and whitish; the other symptoms were, thirst; slight pain on pressure of the epigastrium; meteorism; one liquid stool; an occasional cough; a weak and transient *râle sibilant* on both sides; the pulse full, and of moderate frequency; the skin moist; and the patient said that he had perspired a good deal that morning. The prescriptions were VS. ad ℥xij., a solution of syrup of gum, with twelve grains of chloride of soda; whey; a chloruretted enema, and chloruretted lotions.

Seventh day. The blood taken yesterday is not buffed, and is rather diffluent; the weakness continues, but the headach is less. Yesterday evening there was slight epistaxis; there is no sign of the typhoid eruption; the patient has had one liquid stool; the state of the abdomen is the same.

Eighth day. The eyes are still brilliant, and the skin hot; the pulse has almost lost its frequency; the weakness is still considerable; there has been one loose stool during the last twenty-four hours.

On the following days the patient continued to get better, but very slowly. Although no one of the local symptoms was intense, convalescence did not begin till the seventeenth day; and at an advanced period the patient had a very slight attack of ague, which disappeared under the use of an infusion of cinchona. Some days afterwards he complained of considerable pain in the region of the left kidney, which yielded to the application of ten leeches. He went out after having remained in the hospital six weeks. (P. 348.)

We pass over our author's observations on the diagnosis of typhus fever, partly because we think it is a disease which can rarely be confounded with any other one, and partly because there is nothing very new in his remarks.

*Prognosis.* There are few diseases in which the deaths are so numerous in proportion to the number of patients. Out of 147 cases of typhus admitted into the clinical wards of the Hôtel Dieu, from the beginning of 1828 to the end of 1832, forty-seven died, being nearly one in three. It would appear, however, that the great mortality at the Hôtel Dieu partly depends on its proximity to the *Bureau Central*, in consequence of which it is the receptacle chosen for the worst cases.

Out of seventy-three cases where the attack was sudden, there were twenty-six deaths. Out of thirty-nine cases with premonitory symptoms, there were twenty deaths. On which the Professor says, strangely enough,

"La différence entre ces résultats est assez forte pour ne point être négligée; il est évident que le pronostic est plus fâcheux chez les sujets chez lesquels l'invasion a lieu d'une manière subite, et qu'au contraire, il est plus favorable chez ceux qui ont offert des préludes." (P. 433.)

It is obvious that the reverse is the fact, provided our author's figures are correct.

When a decided amelioration takes place, particularly from the tenth to the twentieth day, and the symptoms return after a short time with greater intensity, the termination of the case is almost always unfavourable.

*Treatment.* In the slighter cases of typhus fever, it is sufficient to prescribe cooling beverages, such as lemonade and orangeade, and linseed poultices to the abdomen, if it is painful; together with lotions of vinegar and water applied over the whole body; or baths, if the heat is great; mucilaginous enemata several times a day; cold compresses to the forehead when there is headach; and hot poultices, or even sinapisms, if there is a tendency to coma.

These remedies alone would generally be sufficient; nevertheless, it is useful, even in the slightest cases, to begin with a bleeding, whose first effect is to diminish the headach, and to hasten the approach of the period when it ceases. When the headach or the abdominal pain is intense, leeches may be applied; in the former case to the mastoid apophyses, in the latter to the anus. Our author treated a large number of cases with the chloride of soda, but, though at first his success was marked, subsequent cases seemed to show that the remedy had no especial efficacy. The majority took from fifty-four to ninety ounces daily of a solution containing a grain or a grain and a half of the chloride in an ounce, besides the use of lotions and enemata impregnated with the same substance.

This work certainly does great honour to Professor Chomel and the illustrious school of medicine to which he is attached; but it must not be considered as a treatise on typhus fever in general, but on the disease as it appeared in Paris from 1828 to 1832; for many of the symptoms mentioned by our author (such as the eruption and the diarrhœa,) cannot be admitted into a definition of the disease, as they by no means universally attend it. The book is certainly a large one; yet those of our readers who are not overwhelmed with occupation will do well to peruse it, for it is the production of a genuine observer, —a man enjoying vast opportunities of gathering knowledge, and possessing talent to profit by them.

